# Metagenomic next-generation sequencing promotes diagnosis and treatment of *Pneumocystis jirovecii* pneumonia in non-HIV infected children: a retrospective study

**DOI:** 10.1186/s12890-024-03135-1

**Published:** 2024-07-12

**Authors:** Zhenyu Zhang, Tingyan Liu, Meixiu Ming, Meili Shen, Yi Zhang, Hanlin Chen, Weiming Chen, Jinhao Tao, Yixue Wang, Jing Liu, Jihua Zhou, Guoping Lu, Gangfeng Yan

**Affiliations:** 1 Pediatric Intensive Care Unit, Children’s Hospital of Fudan University, National Children’s Medical Center, No.399 Wanyuan Rd., Minhang Dist., Shanghai, 201102 China; 2 Medical Department, Nanjing Dinfectome Technology Inc., Nanjing, China; 3 Department of Clinical Epidemiology, Children’s Hospital of Fudan University, National Children’s Medical Center, Shanghai, China

**Keywords:** Pediatric intensive care unit, Bronchoalveolar lavage fluid (BALF), Immunodeficiency, Clinical characteristics, Prognosis

## Abstract

**Background:**

Metagenomic next-generation sequencing (mNGS) excels in diagnosis of infection pathogens. We aimed to evaluate the performance of mNGS for the diagnosis of *Pneumocystis jirovecii* pneumonia (PJP) in non-HIV infected children.

**Methods:**

Totally 36 PJP children and 61 non-PJP children admitted to the pediatric intensive care unit from March 2018 to December 2021 were retrospectively enrolled. Clinical features of PJP children were summarized. 1,3-β-D glucan (BDG) test and bronchoalveolar lavage fluid (BALF) mNGS were used for evaluation of PJP diagnostic performance. Antimicrobial management modifications for PJP children after the mNGS results were also reviewed.

**Results:**

*Pneumocystis jirovecii* was detected in all PJP children by mNGS (36/36), and the sensitivity of mNGS was 100% (95% confidence interval [CI]: 90.26–100%). The sensitivity of BDG was 57.58% (95% CI: 39.22–74.52%). Of the 26 (72.2%) PJP patients with mixed infection, twenty-four (66.7%) were detected by BALF-mNGS. Thirteen patients (36.1%) had their antimicrobial management adjusted according to the mNGS results. Thirty-six PJP children included 17 (47.2%) primary immunodeficiency and 19 (52.8%) secondary immunodeficiency, of whom 19 (52.8%) survived and 17 (47.2%) died. Compared to survival subgroup, non-survival subgroup had a higher rate of primary immunodeficiency (64.7% vs. 31.6%, *P* = 0.047), younger age (7 months vs. 39 months, *P* = 0.011), lower body weight (8.0 kg vs. 12.0 kg, *P* = 0.022), and lower T lymphocyte counts.

**Conclusions:**

The mortality rate of PJP in immunosuppressed children without HIV infection is high and early diagnosis is challenging. BALF-mNGS could help identify PJP and guide clinical management.

**Supplementary Information:**

The online version contains supplementary material available at 10.1186/s12890-024-03135-1.

## Introduction

*Pneumocystis jirovecii* pneumonia (PJP) is a severe and life-threatening respiratory infection that primarily affects immunosuppressed patients, with high morbidity and mortality rates [1, 2]. With the increasing number of children undergoing transplantation and immunosuppressive therapy, as well as advancements in the diagnosis of primary immunodeficiencies in children, the incidence of *Pneumocystis jirovecii* pneumonia (PJP) in pediatric patients is gradually rising [3, 4]. Furthermore, the infant population, with its relatively weak and immature immune system, may also be a risk factor for PJP infection. There have been multiple previous studies focusing on the occurrence of PJP following HIV infection, but there has been comparatively less emphasis on PJP in non-HIV infected children, particularly regarding severe PJP cases [5]. The clinical characteristics and prognosis of these patients are still unclear.

PJP often leads to severe and fatal infections. The prognosis of patients is intricately related to timely diagnosis and appropriate treatment. This is particularly crucial for patients with severe respiratory failure caused by PJP, as early diagnosis and selection of effective anti-infective therapies are the keys to successful rescue efforts. Compared to HIV-infected patients, non-HIV infected patients with PJP typically exhibit a more gradual onset of symptoms, experience a rapid progression of the disease, are at an increased risk of concurrent co-infections with other pathogens, and display a higher frequency of developing respiratory distress syndrome. For these patients, early, rapid, and accurate diagnosis is the key to guiding the treatment and improving prognosis.

Pathogen metagenomic next-generation sequencing (mNGS) is an emerging molecular biology detection technology that provides comprehensive and unbiased detection of nucleic acids of all pathogens and is capable of detecting pathogens in a variety of samples, including bronchoalveolar lavage fluid (BALF) [6]. Pathogen mNGS holds particular value in immunosuppressed patients. Notably, mNGS obviates the need for culture, offering a rapid turnaround time. Furthermore, it enables simultaneously detection of bacteria, viruses, fungi, parasites, and other pathogens [7], irrespective of the presence of antibacterial drugs. Importantly, mNGS excels in characterizing mixed infections, severe infections, as well as identifying rare and novel pathogens, thereby offering additional diagnostic benefits in these challenging scenarios. Pathogen mNGS is highly sensitive and specific in small populations, and simultaneous mNGS of bronchoalveolar lavage fluid and blood samples showed good concordance in patients with *Pneumocystis jirovecii* pneumonia [8]. In this study, we evaluated the diagnostic performance of mNGS in non-HIV infected children with PJP infection. Furthermore, we comprehensively analyzed the clinical characteristics and prognosis of this specific patient population.

## Materials and methods

### Patient enrollment and sample collection

This study is a retrospective study of pneumonia patients admitted to the pediatric intensive care unit (PICU) of Children’s Hospital of Fudan University from March 2018 to December 2021. Enrolled criteria were as follows: (i) non-HIV infected; (ii) with dry cough or fever, shortness of breath; (iii) chest computed tomography (CT) showing multiple ground-glass interstitial exudates, reticular or consolidated shadows in both lungs; (iv) underwent both mNGS and conventional microbiological tests. Patients were excluded if (i) age was less than 28 days or more than 18 years; (ii) HIV infected; (iii) mNGS was not used; (iv) medical record was incomplete; (v) diagnosis and prognosis information was uncertain; and (vi) hospital stay was less than 48 h. Diagnostic criteria for PJP were as follows [9]: (i) blood or BALF BDG tests positive twice; (ii) elevated peripheral blood lactate dehydrogenase (LDH) (> 618 U/L); (iii) *P. jirovecii* trophozoites (and/or cysts) were microscopically identified following Wright–Giemsa staining. (iv) Three and above reads of *P. jirovecii* were detected by BALF-mNGS. The clinical comprehensive diagnosis of PJP or non-PJP was made by two senior expert pulmonary doctors after discussion with the medical team based on clinical symptoms, laboratory findings, chest radiology, microbiological tests and treatment responses. Eventually 36 PJP and 61 non-PJP patients without HIV infection were included in this study.

Patients were considered immunosuppressed if they met at least one of the following criteria [10]: (i) with hematological tumors; (ii) treated with chemotherapy for solid tumors within 28 days; (iii) post-transplantation; (iv) receiving immunosuppressive agents; (v) with primary immunodeficiency; (vi) with agranulocytosis less than 1000/uL; (vii) treated with daily glucocorticoid use ≥ 0.3 mg/kg and days ≥ 14 d (or use equivalent hormone); (viii) having CD4 count < 200 cells/uL or proportion < 14%. Diagnostic criteria for primary immunodeficiency disease was based on the presence of a pathogenic gene, according to the PID classification criteria issued by the International Society of Immunology (IUIS) in 2015 [11]. This non-interventional study was approved by the Research Ethics Committee at the Children’s Hospital of Fudan University (IRB:2019 − 312). All data were de-identified and anonymously processed.

### Clinical evaluations

Relevant clinical information was collected for each patient, including general information (age, weight, sex, critical illness score at admission, days of hospitalization, etc.), conventional microbiological test (CMT) results (including quantitative serum CMV-DNA, serum or BALF BDG, BALF culture, and BALF Wright’s stain), BALF mNGS results, general laboratory test results (including complete blood count, liver and kidney function, and T lymphocyte counts), imaging data (chest CT scan), treatments administered (before and after diagnosis of PJP infection, drug resistance), and status upon transfer out of the PICU (survival vs. non-survival). PJP diagnosis was considered promoted by mNGS with following criteria: (i) identification of new PJP cases through mNGS analysis; (ii) confirmation of clinical suspected PJP diagnoses by mNGS in the absence of prior clinical and molecular evidence.

### Nucleic acid extraction and metagenomic next-generation sequencing

BALF samples were collected according to standard procedures. BALF DNA was extracted using the TIANamp Magnetic DNA Kit (Tiangen, China) according to the manufacturer’s protocol. No template controls (NTCs) were also included in the DNA extraction step. The quantity and quality of DNA were evaluated using Qubit 2.0 Fluorometers and Nanodrop 8000 spectrophotometers (Thermo Fisher Scientific, USA), respectively.

### Library preparation and sequencing

BALF DNA and NTCs DNA were fragmented into 150–300 bp. According to the manufacturer’s protocols, DNA libraries were prepared using the KAPA Hyper Prep kit (KAPA Biosystems). Agilent 2100 was used for quality control, and DNA libraries were sequenced in 75 bp single end on the NextSeq 550Dx platform (Illumina, USA).

Raw sequencing data was splited by bcl2fastq2, and high-quality sequencing data were generated using Trimmomatic by removing low quality reads, adapter contamination, duplicated and short (length < 36 bp) reads. Human host sequences were subtracted by mapping to the human reference genome (hs37d5) using bowtie2. Reads that could not be assigned to the human genome were retained and aligned with the microorganism genome database for microbial identification using Kraken 2, and the species abundance were estimated by Bracken. The microorganism genome database containing genomes or scaffolds of bacteria, fungi, viruses, and parasites were downloaded from GenBank (release 238, www.ftp://ftp.ncbi.nlm.nih.gov/genomes/genbank/).

### Sequencing results interpretation and reporting

We used the following criteria when interpreting the results of mNGS:

1. For *Mycobacterium*, *Nocardia*, and *Legionella pneumophila*, a positive result was defined when a species-specific read count of one or more was detected by mNGS.
2. For bacteria (excluding *Mycobacterium*, *Nocardia*, and *Legionella pneumophila*), fungi, viruses, and parasites, a positive result was defined when a species had at least three non-overlapping reads detected by mNGS.
3. Pathogens detected in the negative NTCs were excluded from the analysis, except when the read counts in the samples were at least 10-fold higher than those observed in the NTCs.

### Statistical analysis

Continuous variables data were expressed as the mean ± SDs or median with interquartile range (IQR). Student’s *t-test* or Kruskal-Wallis test was used to analyze the normally distributed or non-normal distribution data. Categorical variables were expressed as percentages (%), and statistical tests were performed using the Chi-squared test or Fisher’s exact test. Patients were divided into PJP and non-PJP groups based on a comprehensive clinical diagnosis, and the sensitivity, specificity, positive compliance rate and negative compliance rate were calculated for mNGS and BDG methods, respectively. Multivariable logistic regression, controlling for factors such as gender, age, weight, underlying disease and pediatric logistic organ dysfunction-2 (PELOD-2) score, was used to assess association of mNGS promoted PJP diagnosis and prognosis of PJP patients. All statistical calculations were performed using SPSS 22.0 statistical software (SPSS Inc.). A P value < 0.05 was considered significant.

## Results

### Baseline characteristics

Patient characteristics on admission are summarized in Table S1. A total of 36 non-HIV-infected PJP and 61 non-PJP pediatric patients were included in this study. There were no significant differences in age, weight, and gender compositions between non-PJP and PJP patients. Fever (88.9% vs. 67.2%, *P* = 0.017) and immunodeficiency (100% vs. 47.5%, *P* < 0.001) were more common in PJP group. Children with PJP had lower PELOD-2 scores (*P* < 0.001). Noticeable disparities were observed in various laboratory test indicators, including white blood cell (WBC) count, lymphocyte (LYM) count, neutrophil (NEU) count, C-reactive protein (CRP) levels, and lactic dehydrogenase (LDH) levels, as well as immunological markers (CD3, CD8, CD4, CD4/CD8 ratio), between non-PJP and PJP pediatric patients. The duration of PICU stay, hospital stay, and mechanical ventilation were similar between the PJP group and the non-PJP group. The non-survival rate was relatively higher in children with PJP compared to non-PJP group (44.4% vs. 24.6%, *P* = 0.070).

### Survival outcome analysis in patients with PJP

The patients were divided into survival (19 patients) and non-survival (17 patients) groups according to their status when transferred out of the PICU (Table 1). Compared to the survival group, the non-survival group had a higher proportion of patients with primary immunodeficiency (64.7% vs. 31.6%, *P* = 0.047), younger age (median 7 months vs. 39 months, *P* = 0.011), lower body weight (median 8.0 kg vs. 12.0 kg, *P* = 0.022), and a higher PELOD-2 score when entering the PICU (5 vs. 3, *P* = 0.015). Procalcitonin (PCT) was increased (0.9 ng/mL vs. 0.3 ng/mL, *P* = 0.036) and serum albumin was decreased (31.6 g/L vs. 32.7 g/L, *P* = 0.022) in the non-survival group. In terms of immune function, cell counts of CD3 (154 × 10^6^/ml vs. 340 × 10^6^/ml, *P* = 0.015), CD8 (59 × 10^6^/ml vs. 186 × 10^6^/ml, *P* = 0.004), and CD4 (39 × 10^6^/ml vs. 137 × 10^6^/ml, *P* = 0.032) were significantly lower in the non-survival group.

**Table 1:** Comparison of survival and non-survival groups in children with PJP

Characteristic	Survival (*n* = 19), median (IQR) or N (%)	Non-survival (*n* = 17), median (IQR) or N (%)	*p*-value
Age (month	39(11, 62)	7 (5, 31)	0.011*
Weight (kg)	12.0(8.0, 16.5)	8.0(6.0, 10.0)	0.022*
Gender			0.46
Male	15 (78.9%)	11 (64.7%)	
Female	4 (21.1%)	6 (35.3%)	
Primary immunodeficiency	6 (31.6%)	11 (64.7%)	0.047*
Secondary immunodeficiency	13 (68.4%)	6 (35.3%)	
Use of corticosteroids	13 (68.4%)	13 (76.5%)	0.72
Use of caspofungin	15 (78.9%)	12 (70.6%)	0.71
Co-infection (refer to Table S2)	14 (73.7%)	15 (88.2%)	0.41
PELOD-2 score	3 (3, 5)	5 (4, 7)	0.015*
OI	6 (3, 13)	11 (6, 14)	0.26
Disease-onset to diagnosis time (day)	16 (15, 16)	19 (17, 18)	0.86
Disease-onset to SMZ use time (day)	13 (8, 19)	18 (10, 20)	0.67
Duration of PICU stay (day)	21 (17, 21)	16 (9, 22)	0.35
Duration of hospital stay (day)	34(25, 54)	23(10, 45)	0.11
Duration of mechanical ventilation (day)	9 (6, 20)	11 (6, 18)	0.73
WBC (×10^9^/L)	6 (4, 15)	7 (4, 10)	0.98
LYM (×10^9^/L)	0.6(0.3, 3.3)	0.8(0.5, 1.8)	0.21
NEU (×10^9^/L)	5 (2, 7)	5 (3, 7)	0.76
CPR (mg/L)	17(8, 66)	26(8, 81)	0.59
PCT (ng/ml)	0.3(0.1, 0.6)	0.9(0.2, 1.9)	0.036*
IL-6 (pg/ml)	91(12, 221)	63(26, 189)	0.57
CD3 (×10^6^/ml)	340(191, 1,470)	154(51, 468)	0.015*
CD8 (×10^6^/ml)	186(94, 574)	59(15, 105)	0.004*
CD4 (×10^6^/ml)	137(61, 549)	39(16, 98)	0.032*
CD4 ratio (%)	27(11, 43)	18 (3, 23)	0.13
CD4/CD8	0.9(0.3, 1.9)	1.1(0.8, 2.2)	0.96
LDH (U/L)	633(493, 843)	802(511, 1,293)	0.28
LaC (mmol/L)	1.1(0.8, 1.7)	1.3(0.9, 2.2)	0.2

*Note* PELOD-2: Pediatric logistic organ dysfunction-2; IQR: Interquartile range; OI: Oxygenation Index; WBC: White blood cell; LYM: Lymphocyte; NEU: Neutrophil; CRP: C-reactive protein; PCT: Procalcitonin; IL-6: Interleukin-6; LDH: Lactic Dehydrogenase; LaC: Lactate

### Comparison of characteristics of primary and secondary immunodeficiency in patients with PJP

**Fig. 1:**
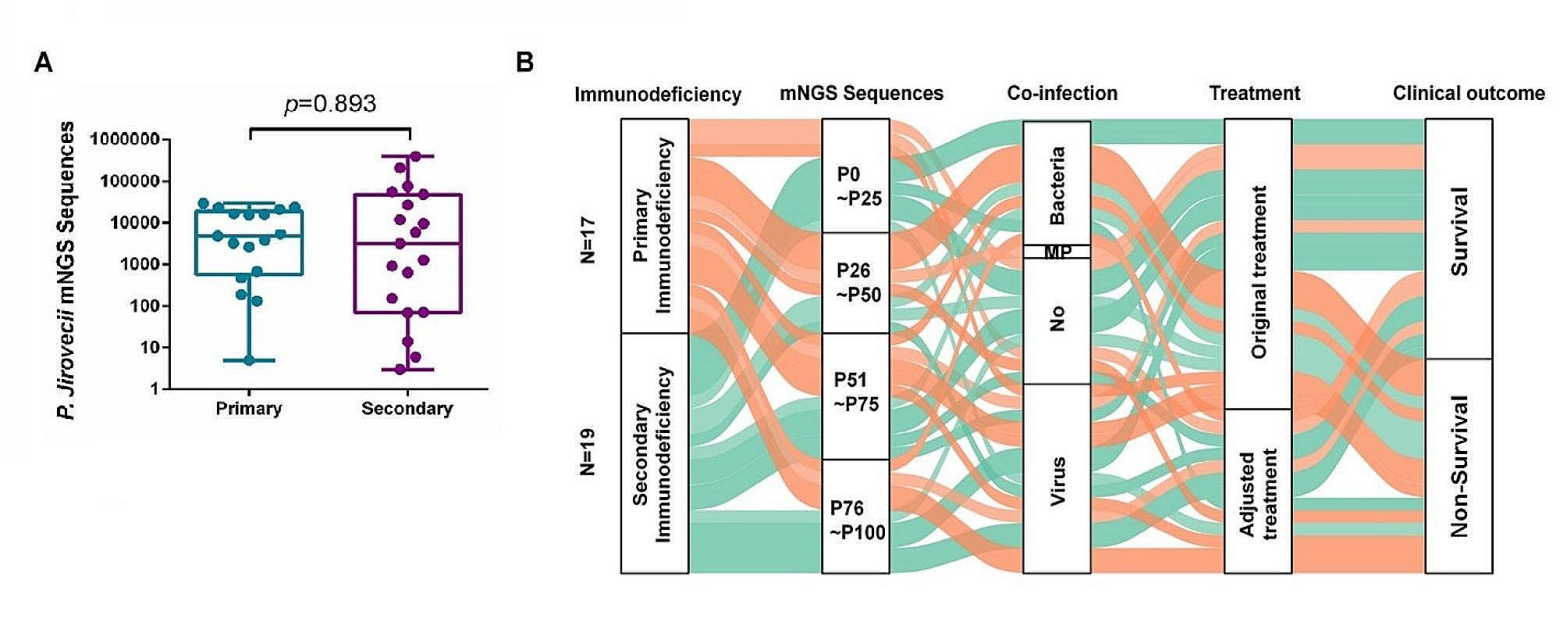
Comparison of characteristics of primary immunodeficiency and secondary immunodeficiency diagnosed with PJP. (**A**) *P. jirovecii* mNGS sequences of PJP in primary and secondary immunodeficiency children. (**B**) A Sankey diagram showing the dynamics of clinical courses in primary and secondary immunodeficiency groups children. To generate the Sankey diagram, we collected data of mNGS sequences (showed with interquartile range), diagnosis, treatments and outcomes

Immunodeficiency is an essential factor in *Pneumocystis jirovecii* infection. To gain deeper insights into the contribution of host immune function in the pathogenesis of the disease, we categorized patients into primary immunodeficiency and secondary immunodeficiency groups based on their underlying disease background (Table 2). Compared with children with secondary immunodeficiency, children with primary immunodeficiency were younger (6 months vs. 39 months, *P* < 0.001) and had lower weight (7.0 kg vs. 12.5 kg, *P* = 0.005). The time to the diagnosis of PJP (22 days vs. 13 days, *P* = 0.048) and the time from onset to initiation of TMP-SMZ (20 days vs. 10 days, *P* = 0.007) were significantly longer in the primary immunodeficiency group. Laboratory test results found that the CD4/CD8 ratio was higher in patients with primary immunodeficiency disease (1.8 vs. 0.6, *P* = 0.022). No significant differences were observed in the number of *P. jirovecii* reads detected by mNGS between the two groups (Fig. 1A). Compared with children with secondary immunodeficiency, children with primary deficiency were more likely to be infected with other pathogens (82.4% vs. 63.2%, *P* = 0.199). A Sankey diagram displays the disease course of the two groups of children, and the results suggest that there is still a certain degree of heterogeneity in the two groups of children (Fig. 1B).

**Table 2:** Comparison of primary and secondary immunodeficiency groups in children with PJP

Characteristic	Primary immunodeficiency(*n* = 17), median (IQR) or N (%)	Secondary immunodeficiency(*n* = 19), median (IQR) or N (%)	*p*-value
Age (month)	6 (4, 7)	39(26, 60)	< 0.001*
Weight (kg)	7.0(5.5, 8.5)	12.5(10.5, 16.2)	0.005*
Gender			0.72
Male	13 (76.4%)	13 (68.4%)	
Female	4 (23.5%)	6 (31.6%)	
Use of corticosteroids	10 (58.8%)	16 (84.2%)	0.14
Use of caspofungin	12 (70.6%)	15 (78.9%)	0.71
Co-infection (refer to Table S2)	14 (82.4%)	12 (63.2%)	0.199
PELOD-2 score	5 (3, 7)	4 (3, 6)	0.44
OI	9 (5, 14)	8 (3, 24)	0.33
Disease-onset to diagnosis time (day)	22 (14, 23)	13 (10, 13)	0.048*
Disease-onset to TMP-SMZ use time (day)	20 (25, 26)	10 (7, 17)	0.007*
Duration of PICU stay (day)	17 (11, 12)	20 (10, 27)	0.51
Duration of hospital stay (day)	34(16, 55)	31(20, 51)	0.67
Duration of mechanical ventilation (day)	10 (6, 18)	13 (4, 28)	0.42
Non-survival	11 (64.7%)	6 (31.6%)	0.047*
WBC (×10^9^/L)	8 (5, 15)	5 (4, 9)	0.38
LYM (×10^9^/L)	1.4(0.5, 2.3)	0.5(0.3, 1.5)	0.77
NEU (×10^9^/L)	5 (3, 7)	4 (2, 7)	0.41
CPR (mg/L)	8 (8, 26)	35(12, 100)	0.42
PCT (ng/ml)	0.2(0.1, 1.2)	0.4(0.2, 0.8)	0.36
IL-6 (pg/ml)	26(16, 154)	120(37, 257)	0.37
CD3 (×10^6^/ml)	440(52, 732)	188(148, 331)	0.61
CD8 (×10^6^/ml)	68(18, 278)	120(60, 210)	0.71
CD4 (×10^6^/ml)	82(20, 392)	69(29, 169)	0.51
CD4 ratio (%)	24(3, 43)	23 (23, 25)	0.94
CD4/CD8	1.8(1.0, 3.7)	0.6(0.3, 1.1)	0.022*
LDH (U/L)	741(507, 992)	709(525, 1,088)	0.55
LaC (mmol/L)	1.3(0.9, 2.2)	1.1(0.8, 1.6)	0.26

*Note* PELOD-2: Pediatric logistic organ dysfunction-2; IQR: Interquartile range; OI: Oxygenation Index; WBC: White blood cell; LYM: Lymphocyte; NEU: Neutrophil; CRP: C-reactive protein; PCT: Procalcitonin; IL-6: Interleukin-6;LDH: Lactic Dehydrogenase; LaC: Lactate

### Roles of mNGS in the diagnosis and antimicrobial management of PJP

#### Detection of *Pneumocystis jirovecii* by BALF-mNGS and CMT

Using comprehensive clinical diagnosis as the reference standard, the diagnostic performances of mNGS and BDG were presented in Table 3. The sensitivity and positive predictive value (PPV) of mNGS were 100% (95% CI: 90.26–100%). The specificity and negative predictive value (NPV) of mNGS were 100% (95% CI: 94.13–100%) The specificity and specificity of BDG were 57.58% (95% CI: 39.22–74.52%) and 85.45% (95% CI: 76.64–93.50%), respectively. The PPV and NPV of BDG were 70.37% (95% CI: 49.82–86.25%) and 77.05% (95% CI: 64.50–86.85%), respectively.

**Table 3:** Diagnostic results of mNGS and BDG in patients with PJP

Methods	PJP	Non-PJP	Sensitivity, % (95%CI)	Specificity, % (95%CI)	PPV, % (95%CI)	NPV, % (95%CI)
mNGS +	36	0	100(90.26–100)	100(94.13–100)	100(90.26–100)	100(94.13–100)
mNGS-	0	61				
BDG+	19	8	57.58(39.22–74.52)	85.45(76.64–93.50)	70.37(49.82–86.25)	77.05(64.50-86.85)
BDG-	14	47				

*Note* mNGS: Metagenomic next-generation Sequencing; PJP: *Pneumocystis jirovecii* pneumonia; BDG: 1,3-β-D glucan

**Fig. 2:**
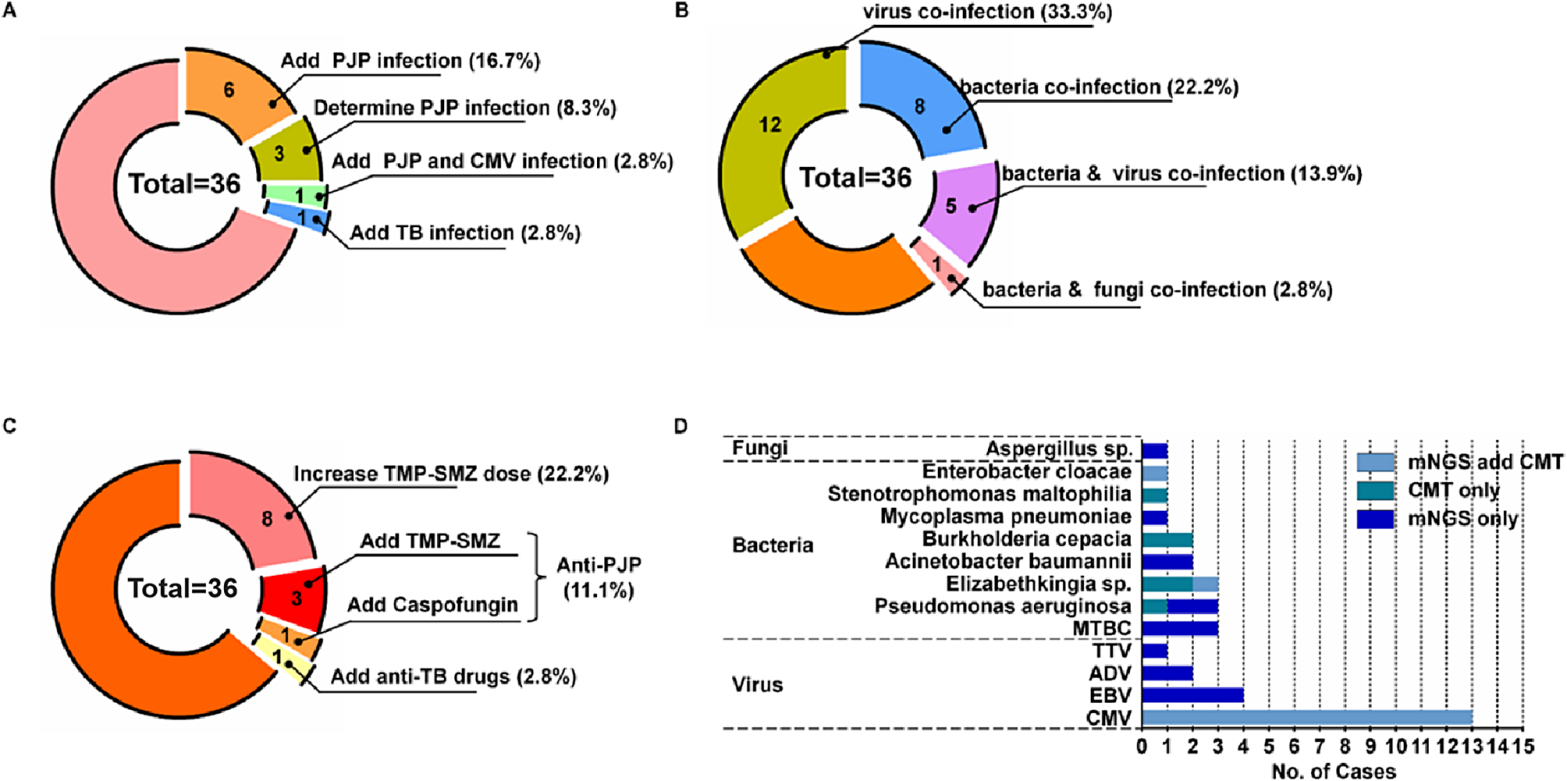
BALF-mNGS roles in pathogen detection and anti-infection decision in PJP patients. (**A**) mNGS promoted PJP decision. (**B**) Mixed infections and co-pathogens identified by mNGS and CMT in 36 PJP patients. (**C**) Anti-infection adjustment based on mNGS results. (**D**)The distribution of other detected pathogens of all PJP patients by mNGS and other methods. PJP: Pneumocystis jirovecii pneumonia; CMT: Conventional microbiological test; TB: Mycobacterium tuberculosis; CMV: Human betaherpesvirus 5; BALF: Bronchoalveolar lavage fluid; mNGS: Metagenomic next-generation sequencing; TMP-SMZ: Trimethoprim-sulfamethoxazole; MTBC: Mycobacterium tuberculosis complex; ADV: Atadenovirus; EBV: Human gammaherpesvirus 4

Among the 36 patients with confirmed PJP, the results of all diagnostic tests are shown in Fig. 2. All patients (36/36) had specific *P. jirovecii* sequences detected by mNGS, with a median number of specific sequences of 4034.5 (IQR: 152.5–20,082); whereas only 52.8% (19/36) of patients were BDG-positive. Ten of 36 patients (27.8%) had a new (seven patients) or confirmed (three patients) diagnosis of *P. jirovecii* infection based on mNGS results; One patient had an increased diagnosis of *Mycobacterium tuberculosis* (TB) infection based on mNGS results (Fig. 2A).

### Mixed infections and co-pathogens of PJP detected by mNGS and CMT

After a comprehensive evaluation by CMT and mNGS, twenty-six (72.2%) cases of PJP patients were identified as mixed infections (Fig. 2B), twenty-four of which (66.7%) were detected by BALF-mNGS. Among the 26 cases of mixed infections, twelve cases (33.3%) were accompanied by viral infections, eight cases (22.2%) were accompanied by bacterial infections, five cases (13.9%) involved additional viral and bacterial infections, and one case exhibited co-occurrence of bacterial and fungal infections (Fig. 2B). Cytomegalovirus (CMV) (13/36, 36.1%) was the most common co-infected virus in children with PJP (Fig. 2B, D; Table S2).

### Clinical value of mNGS for the antimicrobial management and prognosis of children with PJP

A total of 13 patients (36.1%) had their antimicrobial management plans adjusted according to the mNGS results, of which four patients (11.1%) started anti-PJP drugs (three patients received TMP-SMZ, one patient received caspofungin) after mNGS results, eight patients (22.2%) who were already taking anti-PJP drugs had their dose of TMP-SMZ escalated according to the mNGS results. The remaining one case had a new TB diagnosis based on the mNGS results and added anti-TB drug treatments afterward (Fig. 2C). Multivariate logistic regression analysis showed that promoted PJP diagnosis by mNGS is an independent favorable factor for prognosis (*p* = 0.028; OR = 0.03; 95% CI = 0.01–0.68. Table S3).

## Discussion

This study represents a large application of mNGS in diagnosing PJP of non-HIV-infected children including 36 positive cases. While the maximum population previously reported of mNGS application in non-HIV-infected PJP children was 17 cases [15]. The characteristics between subgroup of PJP children without HIV infections were compared to explore potential difference clinical markers.

*P. jirovecii* is the most common opportunistic pathogen in immunosuppressed populations [17]. Previous studies have primarily concentrated on patients infected with HIV. However, the incidence of *P. jirovecii* in this group has declined in recent years due to the introduction of pneumocystis prophylaxis with trimethoprim-sulfamethoxazole in 1989, which has been proven to be highly effective in both the primary prevention and treatment of PJP [25, 29]. The latest epidemiological statistics for 2022 suggest that the incidence of PJP in the United States decreased from 6.7% in 2002 to 3.5% in 2014 [24].Nonetheless, in patients without HIV infection-but with otherwise immune compromised factors-the incidence of PJP has gradually increased [13]. Pediatric population with underlying diseases can also serve as hosts for *P. jirovecii* and develop PJP infection when their immune status is unstable [14]. Apart from the differences in the incidence rate, the clinical course of PJP also differs between HIV-infected and non-HIV-infected individuals. In non-HIV-infected patients, PJP is a fulminant disease associated with a high risk of respiratory failure and mortality [30], while the clinical manifestations of PJP in non-HIV-infected children are often more insidious, and diagnosis is easily missed or delayed [31].

The early diagnosis and treatment of PJP are closely intertwined with the risk assessment. In this study, the included children were divided into survival and non-survival groups to explore the risk factors associated with mortality in cases of PJP infection. Previous adult studies have reported that combined bacteremia, elevated BUN, underlying lung diseases, older age, [higher/lower] white blood cell count, and low lymphocyte count are indicators for a poor prognosis [31–33]. In this study, it was found that the age and weight were statistically lower in the non-survival group as compared to the survival group, while critical PELOD-2 scores were significantly higher in the non-survival group. A comparison was also conducted between children with primary and secondary immunodeficiency. The results indicated that the children with primary immunodeficiency were statistically younger than children identified with secondary immunodeficiency, while the mortality rates were significantly higher in children with primary immunodeficiency. This study also provided evidence that the time between the onset of infection and the subsequent diagnosis and administration of TMP-SMZ in children with primary immunodeficiency was significantly longer than in patients with secondary immunodeficiency.

The diagnosis of primary immunodeficiency can pose challenges due to its inherent genetic nature. Although infections are common, they are more likely to be attributed to more commonly encountered pathogens, and *P. jirovecii* is not often considered as a causative agent. Therefore, early identification of PJP infection and use of TMP-SMZ is crucial for children with primary immunodeficiency. Furthermore, in cases where PJP infection is detected in infant and young children, it is necessary to contemplate the potential presence of primary immunodeficiency after ruling out secondary immunodeficiency and other relevant conditions.

Data focusing on non-HIV-infected patients are currently minimal and inconsistent, however, a large part of this unavailability may be attributable to testing methods. Traditional diagnostic methods for *P. jirovecii* include direct or indirect immunofluorescence assays under a microscope and routine staining (e.g., hexamine silver staining). Compared to HIV-infected individuals, non-HIV-infected individuals have lower fungal burdens, which decrease the sensitivity of microscopy [19, 34]. In HIV-infected patients with PJP, serum [1, 3] β-D-glucan has a high sensitivity, representing that a negative results suggest that the possibility of PJP infection is minimal in individuals with a pretest probability categorized as low or moderate for the presence of the disease [18]. A cut-off value would be beneficial when necessary and can be used in combination with other diagnostic techniques to increase the specificity and sensitivity of the diagnosis [16].

Compared to the traditional detection methods, mNGS emerges as an effective tool for the early diagnosis of PJP, which can detect multiple pathogens simultaneously with high throughput [35], and offer the added advantage of facilitating translation of findings into meaningful alterations in clinical management. Historically, the diagnosis of PJP has been based on traditional detection methods, which lack sensitivity and specificity and often lead to underdiagnosis of PJP in non-HIV-infected children. In relevant studies published to date, mNGS has demonstrated a high sensitivity and a high specificity for PJP in non-HIV-infected patients [12, 15, 19, 22–36]. In this study, the sensitivity of mNGS for PJP in children without HIV infection was 100%, which was similar to previous findings in pediatric patients with PJP [15], as well as those in adult patients with PJP [8, 12]. The sensitivity of BDG in this study was 57.58% and was lower than reported 86.7% in pediatric patients with PJP [15]. It may be due to differences in the positive threshold of BDG test in the studies. BDG > 95 ng/L was defined as positive in this study while in the other study the positive threshold of BDG was 80 ng/L. Future studies of mNGS reads and BDG positivity thresholds could also further assist in the clinical determination of *P. jirovecii* infection and colonization, as the work done by Liu et al. [21].The application of mNGS can expedite the precise diagnosis of PJP, facilitating timely adjustments and optimization of subsequent treatment strategies. In this study, a total of 13 patients (36.1%) had modifications made to their antimicrobial regimens based on the results obtained from mNGS. Specifically, four patients (11.1%) were prescribed anti-PJP medications, while eight patients (22.2%) had escalated SMZ-TMP dosage. mNGS has the additional benefit of detecting multiple types of pathogens parallelly, including bacteria, viruses, and fungi, and shows an superior performance in detecting and describing pathogens that are rare or difficult to detect [27, 37]. In this study, twenty-six children with PJP presented concurrent infections. Notably, mNGS analysis successfully detected 24 of these co-infections, emphasizing the diagnostic efficacy of mNGS in identifying and characterizing mixed infections. This study revealed a substantial occurrence of PJP co-infections, with a notable prevalence of viral co-infections (47.2%) and bacterial co-infections (38.9%). CMV was the most common co-infected virus, accounting for the 36.1% (13/36) of cases. This is consistent with previously reported mNGS-detected PJP co-infections in adults and children, which included mainly viruses and bacteria [12], with CMV being the most common PJP co-infected virus [12, 38–41].

The limitations of this study are its single-center experience, still a small sample size, and retrospective nature. Future investigations with large PJP cohort size are necessary to further explore the clinical characteristics and microbial flora variations in non-HIV infected children with PJP. The fact that PCR and Gomori methenamine silver staining of *P. jiroveci* have not been routinely performed in the laboratory also limited our study to obtain data on its detection performance. Despite the advantages of mNGS in terms of sensitivity and its role in guiding the adjustment of anti-infection treatment, as well as the controlled turnaround time of within 48 h or even 24 h, the interpretation of mNGS results remains challenging. A significant issue is the distinction between pathogenicity and colonization or contamination. A positive mNGS result does not necessarily indicate PJP; the patient may merely be colonized, with pulmonary symptoms stemming from another respiratory infection, given that PJP symptoms are non-specific. Therefore it requires a comprehensive diagnosis of the infectious agents combining clinical features, patient risk factors, various laboratory tests, and multiple pathogenetic test results.

## Conclusions

We found that non-HIV infected pediatric patients with PJP are at a higher risk of mortality and more common occurrence of delays in definitive diagnosis and initiation of antimicrobial therapy, particularly among those with primary immunodeficiency. BALF-mNGS can facilitate the identification of a provisional diagnosis of PJP and provide valuable guidance for clinical management.

### Electronic supplementary material

Below is the link to the electronic supplementary material.

Supplementary Material 1

## Data Availability

The datasets generated and/or analysed during the current study are not publicly available due to institutional regulations on restrictions on disclosure of health information but are available from the corresponding author on reasonable request.

## Abbreviations

PJP: *Pneumocystis jirovecii* Pneumonia
mNGS: Metagenomic Next-Generation Sequencing
HIV: *Human immunodeficiency* Virus
BALF: Bronchoalveolar Lavage Fluid
PICU: Pediatric Intensive Care Unit
BDG: 1,3-β-D Glucan
CMT: Conventional Microbiological Test
WBC: White Blood Cell
LYM: Lymphocyte
NEU: Neutrophil
LDH: Lactic Dehydrogenase
LaC: Lactate
CRP: C-Reactive Protein
PELOD-2: The Pediatric Logistic Organ Dysfunction (Pelod) With Its Updated Version
OI: Oxygenation Index
IL-6: Interleukin-6
CMV: *Human betaherpesvirus* 5
TB: *Mycobacterium tuberculosis*
EBV: *Human gammaherpesvirus* 4
MTBC: *Mycobacterium tuberculosis* Complex
ADV: *Atadenovirus*
TMP -SMZ: Trimethoprim-Sulfamethoxazole
NTCs: No Template Controls
