# Supplementary material for: Metagenomic next-generation sequencing promotes diagnosis and treatment of Pneumocystis jirovecii pneumonia in non-HIV infected children: a retrospective study
Source: BMC Pulm Med. 2024 Jul 12;24:338. doi: 10.1186/s12890-024-03135-1 (PMC11241876; doi:10.1186/s12890-024-03135-1)
Supplement: Supplementary file 1 — Supplementary Material 1 [file 12890_2024_3135_MOESM1_ESM.docx]

**Supplementary Materials**

**Metagenomic next-generation sequencing** **promotes diagnosis and treatment of *Pneumocystis jirovecii* pneumonia in non-HIV infected children**

**Running title:** mNGS improves *P. jirovecii* pneumonia diagnosis and treatment

**Authors：**

Zhenyu Zhang^1†^, Tingyan Liu^1†^, Meixiu Ming^1†^, Meili Shen^2^, Yi Zhang^3^, Hanlin Chen^2^, Weiming Chen^1^, Jinhao Tao^1^, Yixue Wang^1^, Jing Liu ^1^, Jihua Zhou^1^, Guoping Lu^1*^ and Gangfeng Yan^1*^

**Affiliations:**

^1^ Pediatric Intensive Care Unit, Children’s Hospital of Fudan University, National Children’s Medical Center, Shanghai, China

^2^ Medical Department, Nanjing Dinfectome Technology Inc., Nanjing, China

^3^Department of Clinical Epidemiology, Children’s Hospital of Fudan University, National Children's Medical Center, Shanghai, China

**†These authors have contributed equally to this work.**

***Co-correspondence.**

*** Correspondence to:**Guoping Lu, MD and Gangfeng Yan, MD

Pediatric Intensive Care Unit,

Children’s Hospital of Fudan University, National Children’s Medical Center,

No.399 Wanyuan Rd., Minhang Dist., Shanghai, 201102, China

Email: 13788904150@163.com and gangfeng_yan@fudan.edu.cn.

Table S1: Baseline characteristics of enrolled patients

|  | **PJP(n=36), median (IQR) or N (%)** | **non-PJP(n=61), median (IQR) or N (%)** | ***p-value*** |
| --- | --- | --- | --- |
| **Gender** |  |  | 0.14 |
| **Male** | 26 (72.2) | 35 (57.4) |  |
| **Female** | 10 (27.8) | 26 (42.6) |  |
| **Age(month)** | 17.0 (6.0-41.0) | 35.0 (8.0-66.0) | 0.11 |
| **Weight(kg)** | 9.8 (7.0-13.8) | 12.0 (8.0-19.5) | 0.14 |
| **Fever** |  |  | 0.017* |
| **No** | 4 (11.1) | 20 (32.8) |  |
| **Yes** | 32 (88.9) | 41 (67.2) |  |
| **PELOD-2 score** | 4.0 (3.0-6.5) | 3.0 (1.0-4.0) | <0.001* |
| **Underlying disease** |  |  | <0.001* |
| **Immunocompetent** | 0 ( 0.0) | 32 (52.5) |  |
| **Primary immunodeficiency** | 17 (47.2) | 8 (13.1) |  |
| **Secondary immunodeficiency** | 19 (52.8) | 21 (34.4) |  |
| **Duration of PICU stay(day)** | 19.0 (10.5-34.3) | 19.0 (10.0-35.0) | 0.86 |
| **Duration of hospital stay(day)** | 32.5 (18.0-54.5) | 26.5 (13.0-54.0) | 0.69 |
| **Duration of mechanical ventilation(day)** | 12.0 (6.0-27.0) | 9.0 (5.0-22.0) | 0.43 |
| **WBC (×10^9/L)** | 6.4 (3.8-10.9) | 11.6 (7.5-15.7) | 0.002* |
| **LYM (×10^9/L)** | 0.7 (0.4-2.3) | 2.0 (1.1-3.2) | 0.001* |
| **NEU (×10^9/L)** | 5.0 (2.2-7.1) | 7.0 (4.7-12.4) | 0.010* |
| **CPR (mg/L)** | 21.5 (8.0-87.0) | 3.0 (0.0-39.0) | 0.004* |
| **PCT (ng/ml)** | 0.3 (0.1-1.0) | 0.4 (0.1-1.4) | 0.92 |
| **IL-6** | 65.9 (18.0-200.7) | 30.4 (8.9-72.2) | 0.063 |
| **CD3 (×10^6/ml)** | 249.5 (119.8-678.7) | 985.7 (471.2-1722.6) | <0.001* |
| **CD8 (×10^6/ml)** | 99.2 (24.1-220.7) | 332.4 (192.0-558.1) | <0.001* |
| **CD4 (×10^6/ml)** | 69.6 (27.7-321.1) | 526.7 (301.7-1027.8) | <0.001* |
| **CD4 ratio（%）** | 23.6 (6.8-34.0) | 36.0 (28.2-45.1) | 0.002* |
| **CD4/CD8** | 1.0 (0.5-1.9) | 1.7 (1.2-2.5) | 0.032* |
| **LDH (U/L)** | 728.0 (509.5-1061.0) | 392.0 (304.0-533.5) | <0.001* |
| **LaC (mmol/L)** | 1.2 (0.8-2.0) | 1.1 (0.6-1.6) | 0.29 |
| **Prognosis outcome** |  |  | 0.070 |
| **Survival** | 20 (55.6) | 46 (75.4) |  |
| **Non-survival** | 16 (44.4) | 15 (24.6) |  |

Note: PELOD-2: The pediatric logistic organ dysfunction-2 ; OI: Oxygenation Index; WBC: White blood cell; LYM: [Lymphocyte](C:/Users/Admin/AppData/Local/youdao/dict/Application/8.10.3.0/resultui/html/index.html#/javascript:;); NEU: [Neutrophil](C:/Users/Admin/AppData/Local/youdao/dict/Application/8.10.3.0/resultui/html/index.html#/javascript:;); CRP: C-[reactive](C:/Users/Admin/AppData/Local/youdao/dict/Application/8.10.3.0/resultui/html/index.html#/javascript:;) [protein](C:/Users/Admin/AppData/Local/youdao/dict/Application/8.10.3.0/resultui/html/index.html#/javascript:;); PCT: Procalcitonin; IL-6: Interleukin-6;LDH: Lactic Dehydrogenase; LaC: Lactate.

Table S2. Positive-supported results by mNGS and CMT in 36 PJP patients.

| PJP patient | BALF-mNGS promoted PJP decision | | Co-infecton pathogen（co-pathogens NO. excluding *P. jirovecii* ） | BALF-mNGS supported pathogens and unique reads No. | Serum 1，3-β-D Glucan（BDG）（pg/ml） | Culture supported pathogens | Anti-infection adjustment |
| --- | --- | --- | --- | --- | --- | --- | --- |
| P1 | Y, add PJP infection | None (0) | | *P. jirovecii* 11879 | 145.1 | None | Add TMP-SMZ |
| P2 | Y, add PJP infection | None (0) | | *P. jirovecii* 69 | 454.6 | None | N |
| P3 | Y, add PJP infection | *Pseudomonas aeruginosa* (1） | | *P. jirovecii* 20839, *Pseudomonas aeruginosa* 9 | 1522 | None | Increase TMP-SMZ dose |
| P4 | N | *Elizabethkingia meningoseptica* (1) | | *P. jirovecii* 189 | 60 | *Elizabethkingia meningoseptica* | N |
| P5 | Y, add PJP infection | None (0) | | *P. jirovecii* 133 | 23.8 | None | Increase TMP-SMZ dose |
| P6 | Y, add PJP and CMV infection | CMV，*Pseudomonas aeruginosa* (2) | | *P. jirovecii* 23211, CMV 2141; *Pseudomonas aeruginosa* 65 | 238.6 | None | Add TMP-SMZ |
| P7 | Y, determine PJP infection | None (0) | | *P. jirovecii* 5955 | 976 | None | N |
| P8 | N | *Acinetobacter baumannii* (1) | | *P. jirovecii* 152, *Acinetobacter baumannii* 1101 | 1032.7 | None | N |
| P9 | N, add TB infection | CMV, MTBC (1) | | *P. jirovecii* 23971, CMV 444, MTBC 7 | 988.2 | None | Add anti-TB drugs |
| P10 | N | *Mycoplasma pneumoniae, Pseudomonas aeruginosa* (2) | | *P. jirovecii* 671, *Mycoplasma pneumoniae* 1671 | 478.6 | None | N |
| P11 | Y, determine PJP infection | None (0) | | *P. jirovecii* 70 | 985.9 | None | N |
| P12 | N | *Enterobacter cloacae* (1) | | *P. jirovecii* 6, *Enterobacter cloacae* 93 | 731.2 | *Enterobacter cloacae* | N |
| P13 | Y, determine PJP infection | *Elizabethkingia* sp., ADV12 (2) | | *P. jirovecii* 5339, *Elizabethkingia anophelis* 5881, ADV 12 439 | 76.3 | *Elizabethkingia* sp. | N |
| P14 | N | None (0) | | P. jirovecii 631 | 160.6 | None | N |
| P15 | N | CMV, *Torque teno virus* 15 (2) | | *P. jirovecii* 14, *Torque teno virus* 15 14, CMV 14 | 210.2 | None | Increase TMP-SMZ dose |
| P16 | N | CMV (1) | | *P. jirovecii* 29295, CMV 3693 | 69.3 | None | Increase TMP-SMZ dose |
| P17 | Y, add PJP infection | CMV, EBV (2) | | *P. jirovecii* 55310, EBV 17493, CMV 295 | 618.5 | None | Increase TMP-SMZ dose |
| P18 | N | *Burkholderia cepacia*, ADV-B (2) | | *P. jirovecii* 3198, ADV-B 39 | 75 | *Burkholderia cepacia* | N |
| P19 | N | None (0) | | *P. jirovecii* 2612 | 160.6 | None | Increase TMP-SMZ dose, change q8 to q6 |
| P20 | N | None (0) | | *P. jirovecii* 397158 | 60 | None | N |
| P21 | N | *Acinetobacter baumannii* (1) | | *P. jirovecii* 1285, *Acinetobacter baumannii* 767 | 29.2 | None | N |
| P22 | N | CMV, EBV (2) | | *P. jirovecii* 9593, CMV 3825, EBV 972 | 2268.7 | None | N |
| P23 | N（using anti-TB drugs） | CMV, MTBC (2) | | *P. jirovecii* 5, CMV 6425, MTBC 168 | 64.3 | None | N |
| P24 | N | *Stenotrophomonas maltophilia, Burkholderia cepacia, Aspergillus* sp. (3) | | *P. jirovecii* 3231, *Aspergillus* sp. 16 | 276.5 | *Stenotrophomonas maltophilia, Burkholderia cepacia* | N |
| P25 | N | CMV (1) | | *P. jirovecii* 15892, CMV 1739 | 40 | None | N |
| P26 | N | EBV (1) | | *P. jirovecii* 47679, EBV 1265 | 59.2 | None | Add Caspofungin |
| P27 | N | None (0) | | *P. jirovecii* 17811 | 56.5 | None | N |
| P28 | N | *Elizabethkingia* sp. (1) | | *P. jirovecii* 3 | 37.5 | *Elizabethkingia* sp. | Increase TMP-SMZ dose, change q8 to q6 |
| P29 | N | MTBC (1) | | *P. jirovecii* 359, MTBC 21 | 187.3 | none | No（DIED） |
| P30 | N | CMV (1) | | *P. jirovecii* 5872, CMV 66277 | 380.6 | none | no |
| P31 | N | none (0) | | *P. jirovecii* 14 | 37.5 | none | no |
| P32 | N | CMV (1) | | *P. jirovecii* 154, CMV 2197 | 60.4 | none | Increase TMP-SMZ dose, change q8 to q6 |
| P33 | Y, add PJP infection | CMV (1) | | *P. jirovecii* 4838, CMV 6 | 37.5 | none | Add TMP-SMZ |
| P34 | N | CMV (1) | | *P. jirovecii* 15329, CMV 44606 | 60 | none | N |
| P35 | N | EBV (1) | | *P. jirovecii* 207196, EBV 3367 | 600 | none | N |
| P36 | N | CMV (1) | | *P. jirovecii* 75315, CMV 4247 | 81.6 | none | N |

Note: mNGS: Metagenomic next-generation sequencing; PJP, *Pneumocystis jirovecii* pneumonia; BDG: 1,3-β-D glucan. BALF: Bronchoalveolar lavage fluid; Y: Yes; N: No; CMV: *Human betaherpesvirus 5*; TB: *Mycobacterium tuberculosis*; EBV: *Human gammaherpesvirus 4*; MTBC: *Mycobacterium tuberculosis* Complex; ADV: *Atadenovirus*; TMP-SMZ: Trimethoprim-Sulfamethoxazole.

Table S3 The association of mNGS promoted PJP diagnosis and prognosis of patients with PJP

|  | mNGS promoted PJP diagnosis | Prognosis outcome | | Multivariate model* OR (95%CI) | p |
| --- | --- | --- | --- | --- | --- |
|  |  | Non-survival | Survival |  |  |
| PJP (n=36) | Yes | 2 | 8 | 0.03 (0.01-0.68) | 0.028 |
|  | No | 14 | 12 |  |  |

Note: *logistic regression model adjusted gender, age, weight, underlying disease and PELOD-2 score. OR: Odds ratio; 95%CI: 95% confident interval; mNGS: Metagenomic next-generation sequencing; PJP: *Pneumocystis jirovecii* pneumonia.
